# Wetting of the tarsal adhesive fluid determines underwater adhesion in ladybird beetles

**DOI:** 10.1242/jeb.242852

**Published:** 2021-10-19

**Authors:** Pranav Sudersan, Michael Kappl, Bat-El Pinchasik, Hans-Jürgen Butt, Thomas Endlein

**Affiliations:** 1 Max Planck Institute for Polymer Research, Ackermannweg 10, 55128 Mainz, Germany; 2 School of Mechanical Engineering, Tel Aviv University, Tel Aviv-Yafo 69978, Israel

**Keywords:** Bio-adhesion, Capillary force, Air plastron, Insects, Gecko

## Abstract

Many insects can climb smooth surfaces using hairy adhesive pads on their legs, mediated by tarsal fluid secretions. It was previously shown that a terrestrial beetle can even adhere and walk underwater. The naturally hydrophobic hairs trap an air bubble around the pads, allowing the hairs to make contact with the substrate as in air. However, it remained unclear to what extent such an air bubble is necessary for underwater adhesion. To investigate the role of the bubble, we measured the adhesive forces in individual legs of live but constrained ladybird beetles underwater in the presence and absence of a trapped bubble and compared these with its adhesion in air. Our experiments revealed that on a hydrophobic substrate, even without a bubble, the pads show adhesion comparable to that in air. On a hydrophilic substrate, underwater adhesion is significantly reduced, with or without a trapped bubble. We modelled the adhesion of a hairy pad using capillary forces. Coherent with our experiments, the model demonstrates that the wetting properties of the tarsal fluid alone can determine the ladybird beetles' adhesion to smooth surfaces in both air and underwater conditions and that an air bubble is not a prerequisite for their underwater adhesion. This study highlights how such a mediating fluid can serve as a potential strategy to achieve underwater adhesion via capillary forces, which could inspire artificial adhesives for underwater applications.

## Abstract (precis)

**Summary:** Fluid-mediated adhesion seen in animals such as ladybird beetles allows them to attach to surfaces not just in air but also in underwater conditions.

## INTRODUCTION

The question on how insects and other small animals climb smooth and slippery surfaces has fascinated scientists for the past three centuries (Hooke, 1665; Stork, 1980). We know that such animals are able to adhere by using specialised organs on their feet called adhesive pads. These adhesive pads can generally be described as either ‘smooth’ or ‘hairy’. Several insect orders including earwigs, flies and beetles (Gorb and Beutel, 2001), but also several spiders (Coddington and Levi, 1991) and arboreal lizards (Williams and Peterson, 1982), bear hairy pads. Hairy pads show (1) compliance to rough surfaces due to their lower effective modulus, (2) angle-dependent adhesion due to asymmetric hair geometry and (3) self-cleaning capability (Federle, 2006), which makes them suitable to adhere to most surfaces reversibly. The hairs themselves (setae) can branch into smaller fibrillar units (spatulae) as seen in spiders and lizards but are typically undivided in most insects. The hairs in many insects can, however, exhibit different tip geometries, including discoidal, spatula shaped or pointed tips, and distributed throughout the pad depending on sex or species (Bullock and Federle, 2009). Single seta force measurements revealed that discoidal seta show larger pull-off forces than spatula-shaped or pointed setae (Bullock and Federle, 2011), illustrating the role of hair geometry in adhesion. Insect tarsal hairs secrete an adhesion-mediating fluid (‘wet adhesion’) whereas spiders and geckos rely on their dry hairy pads for attachment (‘dry adhesion’). In the ‘wet adhesion’ case, fluid secretion can enforce adhesion through surface tension and viscous forces (Federle et al., 2002; Langer et al., 2004; Dirks, 2014), while ‘dry adhesion’ relies mostly on van der Waals forces (Autumn et al., 2002).

While most of the studies on insect adhesion focused on terrestrial species, underwater insect attachment is much rarer and has been relatively unexplored. Some aquatic insects such as diving beetles (Chen et al., 2014) or midge larva (Kang et al., 2020 preprint) use suction cups to adhere to surfaces (Ditsche-Kuru et al., 2012; Ditsche and Summers, 2014). However, underwater adhesion mediated by secreted liquids requires the displacement of the water at the interface first and a spreading of the fluid on the substrate. One approach is to use an air bubble around the adhesive organs, similar to the air bubbles many secondary aquatic insects and spiders carry on their body for breathing underwater (Seymour and Matthews, 2013). It has been shown in a study by Hosoda and Gorb (2012) that female terrestrial green dock beetles *Gastrophysa viridula* can attach to surfaces underwater by using such an air bubble. Their naturally hydrophobic tarsal hairs trap the bubble around the pads when being submerged underwater, which de-wets the surface on contact. It has been hypothesised that a combination of capillary forces due to the air bubble and hair secretions within the de-wetted area results in its adhesion underwater. However, it remained unclear whether an air bubble is necessary for adhesion and what, if any, contribution it has to the adhesive force. The oily tarsal adhesive fluid found in insects alone might be sufficient to create the necessary capillary adhesion even without a bubble, given that the fluid remains on the hair tips when submerged. In ladybird beetles, the tip of each seta secretes approximately 1 fl of tarsal adhesive fluid by each step (Peisker and Gorb, 2012). The fluid's chemical composition in green dock beetles was identified to be an oil-containing mixture of mostly long chain hydrocarbons (Geiselhardt et al., 2009) with traces of triglycerides, fatty acids and cholesterol in ladybird beetles *Hemisphaerota cyanea* (Attygalle et al., 2000) and *Epilachna vingtipunctuata* (Ishii, 1987), rendering it immiscible with water.

The goal of this paper was to clarify the current understanding of underwater adhesion seen in terrestrial insects which use hairy pads and secrete an oily fluid for attachment. Specifically, the significance of a trapped air bubble to promote underwater adhesion was investigated. We used the ladybird beetle (*Coccinella septempunctata*) as an animal model to first experimentally measure adhesion force of its individual pads in air and underwater conditions, on both smooth hydrophilic and hydrophobic glass surfaces. Male ladybird beetles were chosen as they possess adhesive pads having mostly flat discoidal tipped hairs, which allow them to show superior adhesion on hard surfaces compared with females (Heepe et al., 2016), and they can also walk underwater. Second, we developed a simple theoretical model considering capillary forces to predict the net adhesion force of a hairy pad under different conditions. The case of underwater adhesion was studied in both the presence and the absence of a trapped bubble, to decouple the bubble's contribution to the insect's adhesion. Finally, we discuss key insights gained from our experiments and model with regard to understanding adhesion in other animals. We hope our study will provide new strategies to design bio-inspired materials that show good adhesive properties in both air and underwater conditions, similar to what has been previously reported for terrestrial beetles (Hosoda and Gorb, 2012).

## MATERIALS AND METHODS

Normal adhesion force measurements on a restrained leg in a live beetle were performed. We focused our study only on a single tarsal adhesive pad of the leg by carefully immobilising it (described below) to prevent any dynamic influence of its claws or other tarsomeres/legs, which might otherwise exist under the beetle's natural walking conditions, influencing its adhesion. We characterised adhesion by the pull-off force during detachment, tested on smooth untreated and fluorinated glass surfaces representing hydrophilic and hydrophobic substrates, respectively. When no water was present, we labelled the mode of contact as ‘in air’. Underwater, measurements were done in both the presence and the absence of a trapped air bubble (‘underwater: bubble’ and ‘underwater: no bubble’, respectively) to investigate the air bubble's role in underwater adhesion. Adhesion forces for each of the labelled contact modes were compared for both substrates.

### Insect preparation

Adult seven-spotted male ladybird beetles, *Coccinella septempuctata* (Linnaeus 1758), were purchased from Katz Biotech (Baruth, Germany). The beetles were housed in a plastic box filled with leaves, twigs and stones at room temperature and 60–80% relative humidity with natural daylight. They were fed with raisins, honey and water *ad libitum*. The beetles on average weighed 34±4 mg and were used within 3 weeks of being housed under the above conditions.

**Fig. 1.**
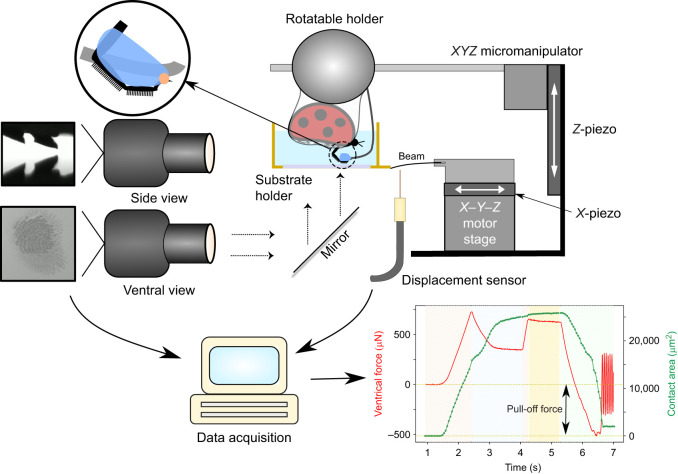
**Adhesion test setup.** Inset (top left) shows a magnified cartoon of the ladybird beetle's (*Coccinella septempuctata*) leg constrained to a solder wire (grey) using Blu Tack (blue) and epoxy glue (orange). The recorded force data and contact area of a distal pad are shown in the bottom-right plot, in which the shaded regions from left to right represent the distinct movement sequence: approach, lateral pull, approach, pause and retract. Negative force values represent attraction, and the minimum force peak during the final retraction step is the adhesion force used for further analysis. Movie 1 shows an animated version of a typical force recording.

An individual beetle was first carefully anaesthetised using small amounts of CO_2_ sublimating from a piece of dry ice and then glued with a small drop of epoxy glue on its elytra to the underside of a heavy steel ball. The ball was held in a bracket, which allowed free rotational movement of the ball in all directions, thus helping to align the suspended beetle over the substrate (see Fig. 1). The bracket with the ball and the beetle could be further positioned by manual micro-manipulators in all three axes before the experiments. The front left leg was carefully fixed at its tibia to a piece of soft solder wire glued to the steel ball, using Blu Tack (Bostik Ltd), allowing us to further align the leg to the substrate. Each leg of a male ladybird beetle has two hairy adhesive pads. For the test, we only allowed the distal pad to make a good contact with the substrate, thus minimising partial or bad contact of the proximal pad. The distal pad was thus restrained by fixing its dorsal side to the wire using Blu Tack. The claws on the leg were also fixed to the wire using epoxy glue to prevent any further movement and to prevent the claws from touching the substrate (Fig. 1, inset). Care was taken to ensure the glue did not contaminate the rest of the tarsomeres. A small piece of non-sticky Teflon tape helped to keep the other legs tucked close to the body and avoided any interference during the adhesion test. After the measurements, the beetle was freed by carefully removing the epoxy glue and Blu Tack from its claws and tibia using a pair of tweezers without harming it, and set free.

### Adhesion test

Adhesion measurements were performed on a custom force measurement setup developed in-house (Fig. 1). A fibre optic displacement sensor (Philtec D20, PHILTEC, Inc.) together with a steel bending beam (spring constant 68.1 N m^−1^) constituted the vertical force sensor. Beam deflection was calibrated using four different known weights (range 2–90 mg) to get the corresponding force (resolution 5 µN). A 3D printed substrate holder (22×22×8 mm) was glued to the end of the bending beam. The holder was designed to enable switching from one substrate to another without removing any glue. It also had transparent side walls which allowed us to fill it with water for the underwater experiments as well as observe the contact. The force sensor was mounted on a stage consisting of an *X*-piezo element, used for precise lateral movements (step size 75 nm). Additionally, a separate *Z*-piezo element (P-629.1CD, Physik Instrumente; resolution 3 nm), fixed upright, was used for vertical up–down motion, bringing the insect in contact with the substrate from the top. Coarse movements of the bottom stage were done using the *XYZ* motors (OWIS GmbH). A coaxial illuminated tube microscope (Navitar) with 2× objective and a stereo-microscope with 1× objective (Wild Heerbrugg) fitted with digital video cameras (Blackfly S, FLIR, 2448×2048 pixels; Basler ace U, 1280×1024 pixels) were used to record the sample contact with the substrate from ventral and side views, respectively. Pad contact area was visualised through the substrate under reflection mode with the help of co-axial illumination. A goniometer was used to adjust the substrate alignment with the ventral view optics to achieve total internal reflection. The data acquisition from the force sensor and cameras, together with the appropriate piezo motion steps, were synchronised using a custom LABVIEW (National Instruments) program. Force data were acquired at a sample rate of 984 Hz, averaged to 512 points per motion step for smoothing. Videos were recorded at 20 frames s^−1^.

The vertical and lateral piezos were used simultaneously to perform approach–retract adhesion tests with the substrate to measure the pull-off force. However, instead of a simple down–up motion, an additional 100 µm lateral sliding motion in the proximal direction was introduced after the leg made contact, to ensure most of the hair tips align well with the substrate (Bullock and Federle, 2009). A further 10 µm compression step (approach) set all hairs in slight compression, which helped maximise the hair contact with the surface. Next, a short pause (1 s) minimised any viscoelastic effects before finally retracting the leg away from the substrate. All approach, retract and lateral slide motions were done at a speed of 62.5 µm s^−1^. Ventral view video recordings were used for contact area extraction while the side view imaging was used to aid in orienting the pad with the substrate before a test.

For underwater experiments, 1 ml Milli-Q water was pipetted into the substrate holder (∼3 mm water level). The beetle (∼5 mm long) was then partially submerged to allow underwater contact of the pad with the substrate (immersion time ∼15 min). In order to achieve contact without a trapped air bubble, the water was first degassed separately in a vacuum chamber at 10 mbar (1 kPa) pressure for 3 h and then pipetted into the holder immediately. The beetle was subsequently immersed, and the trapped air bubble within the pad dissolved into the degassed water in less than 5 min, as verified by the ventral view contact image. Before the experiments, the pad was brought into contact with the clean dry surface 10 times repeatedly (same motion protocol as described above) to ensure the hairs were free of any contaminating particles.

Five force measurements were subsequently performed, each on a fresh spot of the substrate, and were averaged to avoid pseudo-replication during data analysis. Experiments were repeated with distinct male beetles for each combination of contact mode (‘in air’, ‘underwater: bubble’ and ‘underwater: no bubble’) and substrate chemistry (hydrophilic and hydrophobic), using 5 beetles for each combination. Thus, 30 distinct beetles were used in total. After an experiment, the beetle was marked on its elytra and released back into the box to ensure the same beetle was not used for any subsequent adhesion tests.

### Substrate preparation

Standard 20 mm wide glass coverslips were used as the hydrophilic substrate. Glass was wiped with isopropanol, rinsed in water and dried under nitrogen flow before use. For the hydrophobic substrate, the glass coverslip was coated with fluorosilane via chemical vapour deposition. First, the glass was cleaned using isopropyl alcohol. The surface was then plasma cleaned in an oxygen plasma chamber (Femto, Diener Electronic GmbH) for 10 min at 120 W. Next, 0.2 ml of trichloro(1*H*,1*H*,2*H*,2*H*-perfluorooctyl) silane (PFOTS; Sigma Aldrich) was put in a sealed chamber along with the cleaned glass. The chamber was placed under 100 mbar (10 kPa) pressure for 10 min for the chemical vapour deposition process. Finally, the substrate was annealed at 150°C for 3 h. Henceforth, we refer to the hydrophilic untreated glass substrate as simply Glass and the hydrophobic fluorinated glass substrate as PFOTS.

The surface chemistry was characterised by dynamic contact angle measurements, performed with a contact angle goniometer (OCA 35, DataPhysics Instruments GmbH). The substrate's wetting towards a polar (Milli-Q water) and a non-polar (*n*-hexadecane) liquid was tested. Advancing and receding contact angles were measured for a maximum drop volume of 10 µl and with 0.5 µl s^−1^ flow rate (see Supplementary Materials and Methods 2).

### Data analysis

Extraction of pull-off force from force data, image processing, plotting and statistical analysis were all performed in ‘Buggee’, a software tool written in Python using open-source libraries for synchronous analysis of force data and video recordings (https://github.com/PranavSudersan/Buggee).

For measurements in air, the pull-off force was defined as the minimum negative force during the retraction step (Fig. 1, bottom-right plot). For underwater measurements, an additional correction was necessary. When the beetle was partially submerged underwater, the water's contact with the beetle shifted, which influenced the force readout as a result of surface tension and buoyancy. This effect needed to be cancelled. Therefore, ‘background’ force data were recorded, following the exact motion protocol as a typical adhesion test, but where the submerged beetle makes no contact with the substrate. These background data were then subtracted from a typical force curve with substrate contact, by matching the time data, to correct for the external surface tension effects (∼50 µN) for each individual beetle. The pull-off force was subsequently calculated from the minima as before.

Datasets were compared for statistical differences using two-way ANOVA analysis, with contact mode and substrate chemistry as the categorical variables and adhesion force as the dependant variable. Pairwise Student’s *t*-tests were done for *post hoc* analysis and their corresponding *P*-value and Common Language Effect Size (CLES) are reported. Shapiro–Wilk tests were done for each dataset to verify a normal distribution of its residuals and Levene's test was done to check for variance homogeneity, to validate the ANOVA assumptions. Bonferroni's correction was used to account for multiple comparison between groups.

## RESULTS

### Experimental results

**Fig. 2.**
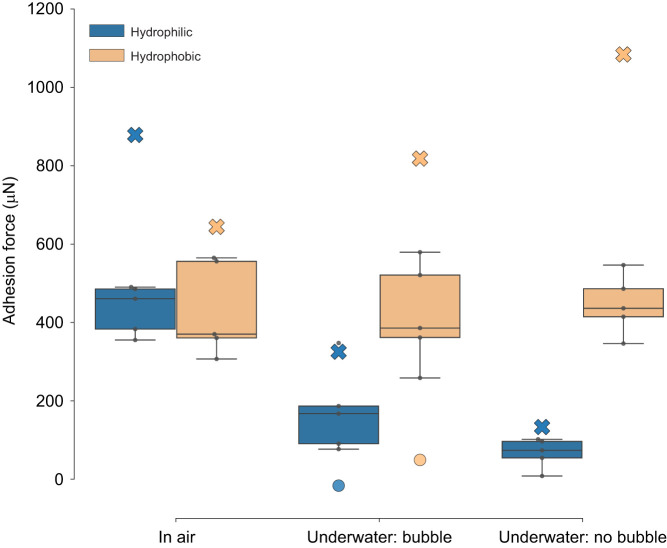
**Adhesion force measurements of a ladybird beetle's distal pad on untreated hydrophilic glass and hydrophobic PFOTS-coated glass substrates in air and underwater conditions.** Box-and-whisker plots show median (horizontal line), upper and lower quartiles (box) and 1.5× interquartile range (whiskers) (*n*=5 per box). The small black circles show the underlying data points. The two modes of contact during underwater experiments are represented separately as ‘bubble’ and ‘no bubble’. Crosses represent theoretical predictions of adhesion force, while circles represent the contribution of the bubble itself, calculated from the capillary bridge model (see Results and Table 1). Two-way ANOVA test showed a significant effect of the contact mode (*P*=0.001, *F*=9.596, d.f.=2) and substrate (*P*<0.001, *F*=36.231, d.f.=1). Significant interaction between these two categories was seen (*P*=0.001, *F*=10.551, d.f.=2). *Post hoc t*-test analysis and linear mixed-effect model results are reported in Supplementary Materials and Methods 3.

In air, adhesion forces of the distal pad of the ladybird beetles against glass and PFOTS were similar, i.e. no significant differences were detected (Fig. 2; Supplementary Materials and Methods 3). In contrast, the underwater adhesion on a PFOTS surface was significantly greater than on glass (*P*<0.001). This stronger adhesion on PFOTS was observed in both the presence and the absence of a trapped bubble. In both cases, the adhesion force reached similar values to those in air. In contrast, on glass, adhesion underwater was significantly reduced when compared with dry conditions, irrespective of the presence of a trapped bubble (*P*≤0.002). In the presence of a bubble, underwater adhesion on glass was slightly higher (CLES=0.84, *P*=0.07).

**Table 1.**
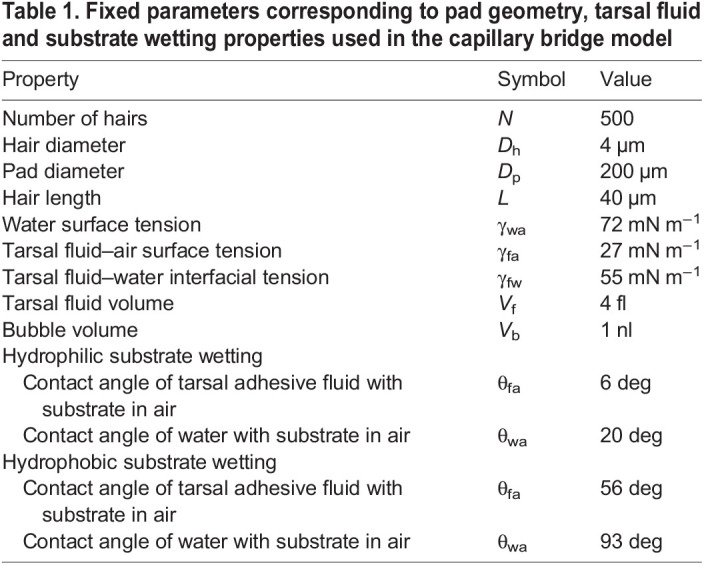
Fixed parameters corresponding to pad geometry, tarsal fluid and substrate wetting properties used in the capillary bridge model

Apart from the three depicted contact modes, we observed an additional fourth mode which occurred in roughly 25% of our underwater experiments (excluded from the above analysis) using degassed water. In this scenario, the ventral view recordings show that none of the hairs appear to contact well with either glass or PFOTS substrate (Movie 2), unlike the other three contact modes (Movie 1). This ‘bad contact’ scenario only happened underwater and showed no adhesion with either substrate. While it was not completely clear why such a contact occurs, there may be two possible reasons. First, the hairs could get bundled as a result of a small air bubble trapped within them which might not have completely dissolved away in the water. The presence of this air–water meniscus could thus lead to elasto-capillary bundling of the hairs, resulting in their disorientation. Second, a thin water layer at the substrate interface might not be drained out to allow the hairs to make contact with the substrate, resulting in a loss of adhesion.

### Theory

#### Capillary bridge model

**Fig. 3.**
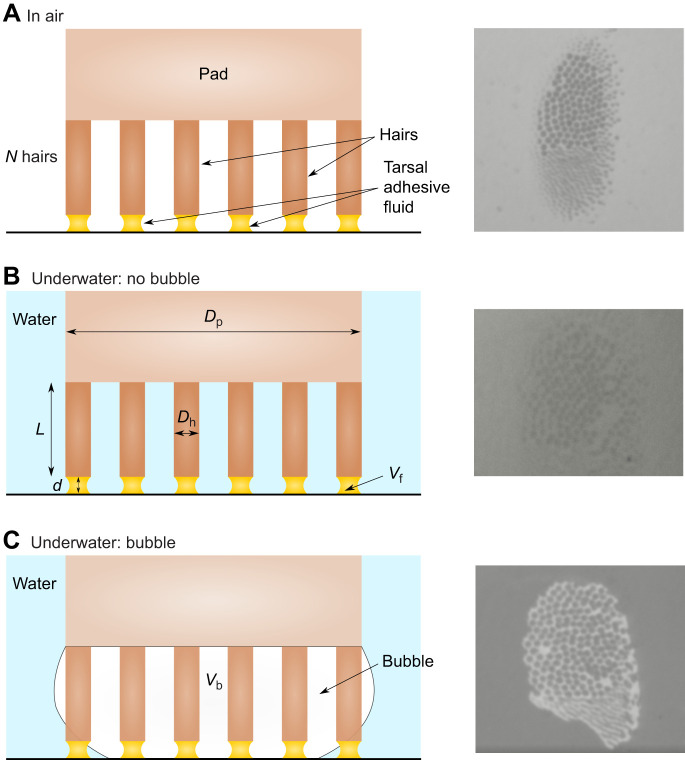
**Capillary bridge model of a hairy adhesive pad.** Left: the hairs make contact with the substrate (hydrophilic or hydrophobic) in three modes: (A) in air, where the tarsal adhesive fluid bridges are surrounded by air; (B) underwater: no bubble, where the fluid bridges are fully surrounded by water; (C) underwater: bubble, where some of the fluid bridges are inside the bubble while others are outside in water (see Results for details). Right: the corresponding ventral view contact images of the beetle's pad seen during adhesion experiments. *L*, hair length; *d*, bridge height; *D*_p_, pad diameter; *D*_h_, hair diameter; *V*_f_, fluid volume; *V*_b_, bubble volume.

The male ladybird beetles used in our experiments are known to possess mostly discoidal hairs on their distal pad. Contact images show that these hair tips are approximately circular (eccentricity ∼0.04), which could allow mechanical pinning of the secreted fluid around its perimeter. Based on this knowledge, we modelled the hairy pad as an array of *N* cylindrical rods of length *L*, and diameter *D*_h_, fixed to a flat circular pad of diameter *D*_p_ (Fig. 3). The hairs and the pad were assumed to be perfectly rigid, for simplicity. The tip of each hair has a tarsal adhesive fluid of volume *V*_f_, mediating contact with the substrate. The fluid is pinned to the circumference of the hair and forms a capillary bridge of height *d*. Similar to our experiments, we considered three modes of contact for the pad: (1) in air, (2) underwater: no bubble and (3) underwater: bubble. In the third case, a bubble of volume *V*_b_ is trapped between the hairs and pinned to the pad circumference (‘Cassie state’).

To characterise the tarsal adhesive fluid and bubble volume, we defined two radii, *s*_f_ and *s*_b_, respectively, by *V*_f_=4/3π*s*^3^_f_ and *V*_b_=4/3π*s*^3^_b_. Here, *s*_f_ and *s*­_b_ are the radii of spheres with equivalent volumes. Fluid and bubble radii were assumed to scale proportional to their corresponding pinned contact diameter. We thus defined the size parameters ϕ_f_=*D*_h_/(2*s*_f_) and ϕ_b_=*D*_p_/(2*s*_b_) for the fluid and bubble, respectively, to conveniently scale their volumes relative to the hair and pad diameters they are pinned to. Larger values of ϕ_f_ and ϕ_b_ represent a smaller volume of liquid (bubble) relative to the hair (pad) that it is pinned to.

The net force of the array *F*_net_ for cases 1 and 2 can be calculated as:(1)
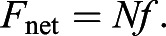
Here, *f* is the capillary force of a single fluid bridge at a distance *d* in air (*f*_air_) or underwater (*f*_water_).

For case 3, the net force is given by:(2)
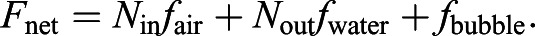
Here, *N*_in_ and *N*_out_ are the number of hairs inside and outside the bubble, respectively, *f*_air_ and *f*_water_ are the capillary forces of the fluid bridge inside and outside the bubble, respectively, and *f*_bubble_ is the capillary force contribution due to the bubble meniscus alone at distance *d*+*L*.

The capillary force *f* is the sum of two contributions: Laplace pressure and surface tension, as given by:(3)
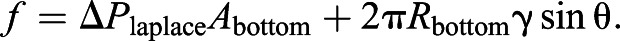
Here, Δ*P*_laplace_ is the Laplace pressure of the equilibrium capillary bridge, θ is the contact angle, *A*_bottom_ is the contact area of the capillary bridge with the substrate at the bottom and *R*_bottom_ is the corresponding radius of contact. Unlike previous analytical treatments (Kim and Bhushan, 2008; Arutinov et al., 2015), force versus distance for a single capillary bridge was calculated by Surface Evolver simulations (Brakke, 1992; De Souza et al., 2008), and used to obtain *F*­_net_ as a function of *d* for each mode of contact (see Supplementary Materials and Methods 1 for details). The adhesion force of the complete hairy pad system was then obtained from the minima of *F*­_net_, where negative force values represent attraction.

We considered *f*_air_ and *f*_water_ to be distinct terms because the capillary force by the tarsal adhesive fluid would be different in air and underwater as a result of its different contact angle and interfacial tension in each case. Using the Young–Dupré equations for each case of fluid–air, fluid–water and water–air interface, one can derive the following relationship for the contact angle of the tarsal adhesive fluid underwater:(4)
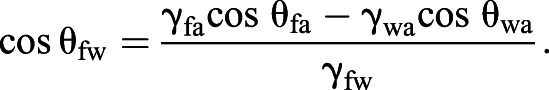
Here, θ_fw_ and θ_fa_ are the contact angles of the tarsal adhesive fluid with the substrate in water and air, respectively, θ_wa_ is the contact angle of water with the substrate in air, γ_fa_ is the surface tension of the tarsal adhesive fluid, γ_wa_ is the surface tension of water and γ_fw_ is the interfacial tension of the tarsal adhesive fluid with water.

Geometric parameters and interfacial properties were kept fixed for all model calculations (Table 1). Here, we assumed the tarsal adhesive fluid to have similar interfacial tension values to *n*-hexadecane (Goebel and Lunkenheimer, 1997). Experimental receding contact angle values for *n*-hexadecane and water on untreated (hydrophilic) and fluorinated (hydrophobic) glass surface were used as θ_fa_ and θ_wa_, respectively (Table S1). Hair and pad geometry, and tarsal fluid volume were assumed to be values typical for a ladybird beetle’s hairy pad (Bullock and Federle, 2009; Peisker and Gorb, 2012).

First, we calculated force–distance curves for a single pinned liquid capillary bridge. Second, the effect of substrate on the force–distance curves of the hairy pad system was compared for each mode of contact. The volume of the bubble would influence its capillary force as well as the proportion of hairs that are inside or outside the bubble. Thus, we also looked at the effect of changing the bubble volume, 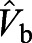, on the net underwater adhesion. Additionally, the influence of varying the hair diameter, *D*_h_, on adhesion was studied for each case, to illustrate the ‘contact splitting’ effect (Arzt et al., 2003).

#### Capillary force of a single liquid bridge

**Fig. 4.**
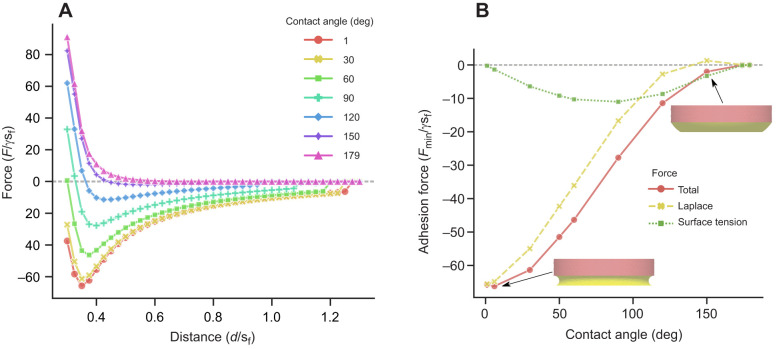
**Simulation of normalised capillary force of a single liquid bridge in contact with a substrate and pinned to a circular perimeter on top.** (A) Force–distance curves for different contact angles of the liquid with the substrate. (B) Adhesion forces, calculated from the minima of the corresponding force–distance curves, plotted as a function of contact angle with the substrate, together with its Laplace and surface tension components (Eqn 3). Simulation snapshots of the liquid meniscus corresponding to 6 and 150 deg angles are depicted. Fluid size parameter, ϕ_­f_=2; negative force values represent attraction. *F*, force; γ, surface tension; *s*_f_, fluid sphere radius; *d*, distance.

Forces due to a single pinned capillary liquid bridge in contact with a substrate were obtained via Surface Evolver simulations (Fig. 4). Generally, the shape of the liquid meniscus determined the strength of its adhesion force. High adhesion (>60% of maximum) was seen for contact angles less than ∼70 deg because of a net negative (convex) curvature of the meniscus, while low adhesion (<10% of maximum) was seen for contact angles greater than ∼150 deg because of the net curvature being close to zero. The Laplace pressure contribution to the net adhesion force dominates for contact angles less than 100 deg (Fig. 4B). Interestingly, its contribution to the adhesion force was mostly non-repulsive for contact angles greater than 90 deg. This is because the low volume of the liquid and its pinned contact line prevent the meniscus from having a high positive (concave) curvature as a result of geometric constraints. Only for a contact angle of 150 deg did the liquid's curvature become positive, manifested in its slightly repulsive Laplace contribution. Surface tension made a significant contribution to the net force only for a small range of contact angles close to 90 deg. For contact angles greater than 150 deg, the net adhesion force approached zero.

The force–distance curves show a general trend of repulsive forces at small distances, a minima at an intermediate distance corresponding to the adhesion force, and finally tending to zero force at large distances until the capillary bridge ruptures (Fig. 4A). The repulsive force seen at small distances is a result of the pinned contact line on the top. A limited volume is available for the liquid to occupy when the gap distance is small, causing the meniscus shape to bulge outwards near the pinned contact line. This creates a net positive curvature, resulting in a positive Laplace pressure and thus repulsion. Without pinning, the capillary forces would have shown high attractive forces on a hydrophilic substrate (De Souza et al., 2008). It is reasonable to expect the contact line to be mechanically pinned around the rim of the discoidal hair tip. As the male ladybird beetle’s pads are majorly composed of discoidal hairs, we proceeded with this assumption to estimate the net adhesion force of the whole pad.

#### Adhesion of a hairy pad: effect of substrate

**Fig. 5.**
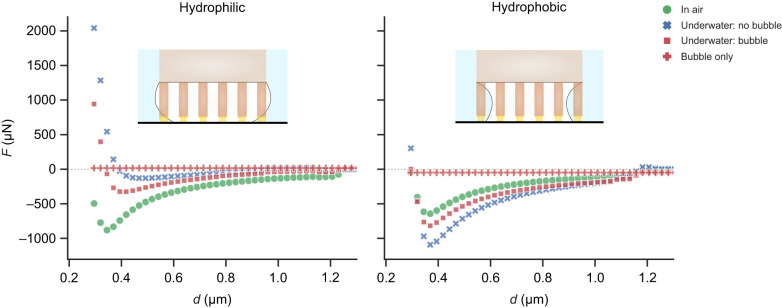
**Theoretical force–distance (*F*–*d*) curves of a hairy pad on a hydrophilic and hydrophobic substrate in air and underwater conditions.** Forces were calculated from the capillary bridge model, with model parameters listed in Table 1. A negative force value represents attraction. The bubble's contribution to the net force for an underwater: bubble contact is denoted by plus symbols. Insets represent the underwater: bubble contact for each substrate.

The force–distance curves of a hairy pad system on a hydrophilic and hydrophobic substrate were predicted based on the capillary bridge model and compared for the different contact modes (Fig. 5). The forces in each case were calculated from Eqns 1 and 2 for fixed geometric and interfacial properties (Table 1).

On the hydrophilic substrate (θ_wa_=20 deg), highest adhesion occurs when the hairs contact in air, while lowest adhesion occurs underwater without a trapped bubble. The presence of a bubble leads to intermediate force values. In contrast, on a hydrophobic substrate (θ_wa_=93 deg), highest adhesion occurs for the underwater case without a trapped bubble – much greater than in air. When a bubble is present, the forces are only slightly larger than in air.

**Fig. 6.**
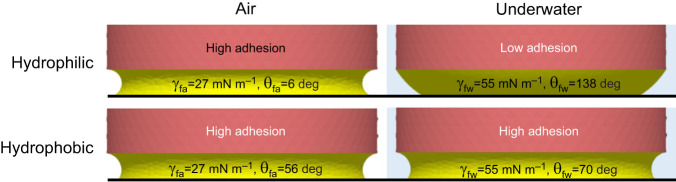
**Simulation snapshots of the oil capillary meniscus in contact with untreated hydrophilic glass and hydrophobic PFOTS-coated glass in air and underwater conditions.** The corresponding interfacial tension (γ) and contact angle (θ) used to predict the ladybird beetle’s adhesion are labelled for each case (fw, fluid–water; fa, fluid–air).

The observed trend in forces can be explained by how the tarsal adhesive fluid wets the surface in each case. On a hydrophilic substrate, the contact angle of the oily fluid is 6 deg when surrounded by air (Table 1) and 138 deg when surrounded by water (Eqn 4). This results in the meniscus shape having net negative and slightly positive curvatures, respectively, resulting in strong adhesion in air and poor adhesion underwater. On a hydrophobic substrate, however, the contact angles of the fluid in air and water are 56 and 70 deg, respectively. In both cases, the contact angles are low, resulting in strong adhesion in both media. Additionally, the interfacial tension of the oily fluid underwater (γ_fw_) is twice that in air (γ_fa_). Thus, there is a higher capillary adhesion for the underwater: no bubble case when compared with the in air case (Fig. 6). Note that as the hair diameter is kept fixed, the observed effects are not a result of changing contact area, but rather of the nature of capillary forces.

The net force in the underwater: bubble case mainly depends on the proportion of hairs inside and outside the bubble (Eqn 2). For the given bubble volume, only some of the hairs make contact with the surface inside the bubble. Therefore, the force curve lies between the in air and underwater: no bubble cases for both substrates.

We observed that the bubble itself does not contribute much to the net force on either substrate (Fig. 5). Its contribution is even slightly repulsive on the hydrophilic substrate as a result of the positive curvature of the bubble, and slightly attractive on the hydrophobic substrate as a result of its negative curvature. This small contribution is manifested by the slightly higher adhesion for the underwater: bubble relative to the in air case for the hydrophobic substrate, as all hairs are within the bubble in this case.

#### Adhesion of a hairy pad: effect of air bubble volume

**Fig. 7.**
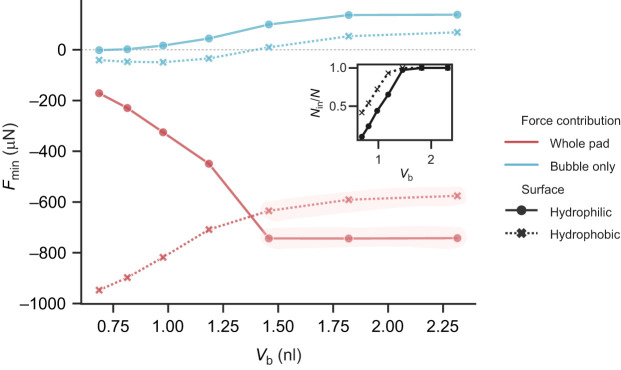
**Adhesion force of a hairy pad as a function of bubble volume (*V*_b_) for the underwater: bubble contact mode.** Adhesion forces (*F*_min_) were calculated from the minima of the respective force–distance curves. Negative force values represent attraction. The inset shows the corresponding fraction of hairs (*N*_in_/*N*) making contact inside the bubble. Highlighted regions represent entrapment of all hairs within the bubble. Remaining model parameters were kept fixed, as listed in Table 1.

The volume of the trapped air bubble can influence its capillary force contribution, as well as change the relative proportion of hairs inside and outside it. To investigate this, we varied the bubble volume, *V*_b_, and compared the maximum adhesion force on both hydrophilic and hydrophobic substrates (Fig. 7). The contribution of the bubble to the net adhesion force is small regardless of its volume, when compared with that of the whole pad (less than 3%). Further, opposite trends of adhesion are seen on the two substrates with changing *V*_b_.

From the previous section, we know that on the hydrophilic substrate, fluid bridges outside the bubble show poor adhesion as a result of the positive curvature of their meniscus. Thus, decreasing *V*_b_ decreases the adhesion force as a consequence of a larger proportion of tarsal hairs being outside the bubble. In contrast, on the hydrophobic substrate, fluid bridges outside the bubble showed higher capillary forces, because of its low contact angle and high interfacial tension in water. Thus, adhesion force increases for a hydrophobic substrate as the bubble size decreases.

A smaller *V*_b_ resulted in increased, but small, attraction by the bubble on both types of substrates. For larger values of *V*_b_, however, the force trend for the whole pad mostly follows that of the bubble. This is because the bubble gets big enough to entrap all the hairs inside it (Fig. 7, inset). Thus, the force contribution due to the fluid bridges remains unchanged, and only the bubble's contribution drives the slight variation in the pad's adhesion at high *V*_b_. Once the bubble is small enough such that some of the fluid bridges start making contact in water, the force trend changes, with a steep decrease (increase) in adhesion force on hydrophilic (hydrophobic) substrate with decreasing bubble volume.

#### Adhesion of a hairy pad: effect of the hair tip diameter

**Fig. 8.**
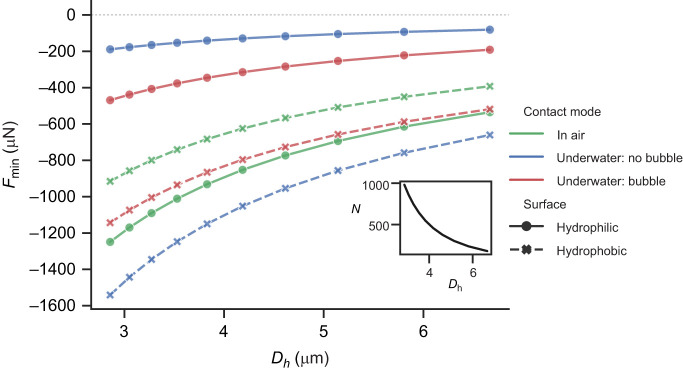
**Adhesion force of a hairy pad on a hydrophilic and hydrophobic substrate as a function of hair tip diameter (*D*_h_).** Volume of each fluid bridge (*V*_f_) scales relative to *D*_h_ based on the parameter ϕ_­f_=2. Total contact area was kept fixed at 6283 µm^2^ throughout. The number of hairs (*N*) varies with *D*_h_, as shown in the inset. Adhesion forces (*F*_min_) were calculated from the minima of the respective force–distance curves, based on the capillary bridge model. A negative force value represents attraction. Remaining model parameters were kept fixed, as listed in Table 1.

The tarsal hairs on a ladybird beetle’s adhesive pad terminate in various shapes, such as discoidal or pointed. We studied this geometric effect on adhesion by changing the hair tip diameter, *D*_h_ (Fig. 8). Here, we fixed the total contact area to 6283 µm^2^ (corresponding to Fig. 5) and varied the number of hairs with *D*_h_ to illustrate the ‘contact splitting’ effect. The tarsal adhesive fluid volume was assumed to scale relative to the hair diameter (ϕ_f_=2). The pad diameter, hair length and bubble volume were kept fixed as per Table 1.

Adhesion force increased with decreasing *D*_h_ for both hydrophilic and hydrophobic substrates in all contact modes. This is consistent with the ‘contact splitting’ theory, which predicts higher adhesion when the contact is split into many small contact points (Arzt et al., 2003). Reducing the hair diameter results in two competing effects: (1) capillary force due to a single fluid bridge decreases as a result of its smaller size and ‘self-similar’ scaling assumption (*f*≈*D*_h_), which decreases the net force; and (2) the total number of fluid bridges increases as the total hair contact area is assumed to be fixed (*N*≈1/*D*_h_^2^), which increases the net force. The second effect dominates, resulting in a higher adhesion force as *D*_h_ decreases.

Similar to the trend in Fig. 5, contact in air shows the highest adhesion force on a hydrophilic substrate for the given range of hair diameters, while on a hydrophobic substrate, the underwater: no bubble contact mode shows highest adhesion. The underwater: bubble contact mode shows intermediate adhesion between the in air and underwater: no bubble contact modes.

## DISCUSSION

Our experiments demonstrate that the ladybird beetle can attach underwater to a hydrophobic substrate even without a bubble trapped around its tarsal hairs. A previous study (Hosoda and Gorb, 2012) proposed that an air bubble is necessary for underwater attachment in terrestrial beetles. However, this is only true for hydrophilic substrates, where a trapped air bubble can facilitate underwater adhesion as a result of the hairs making contact in a de-wetted environment. For a hydrophobic substrate, the adhesion is similar regardless of whether the contact occurs in air or underwater conditions, with or without a trapped bubble. Our theoretical calculations further show that the bubble by itself has a negligible capillary contribution (less than 3%) to the net underwater adhesion of the pad. Direct force measurement of a single similarly sized bubble making contact with a hydrophobic substrate shows a maximum adhesion of less than 50 µN, which further validates that the bubble's contribution is insignificant (see Supplementary Materials and Methods 4).

Predictions of the ladybird beetle’s adhesion from the capillary bridge model agree qualitatively with our experimental results (Fig. 2). In underwater conditions without a trapped air bubble, adhesion to a hydrophobic substrate is significantly larger than to a hydrophilic substrate. This is explained by the different interfacial tension of the oily tarsal secretion and its contact angles with the substrates in air and underwater, which determines the capillary adhesive force in each case (Fig. 6). However, the experiments do not show the predicted ∼1.7 times increase in underwater adhesion relative to that in air on the hydrophobic PFOTS-coated surface. This discrepancy could be due to our assumptions of the oily fluid's interfacial properties, which are not known for the ladybird beetle. Sensitivity analysis of the model does in fact show that the relative adhesion underwater when compared with that in air is sensitive to the fluid's interfacial tension values in air and water (see Supplementary Materials and Methods 5). Direct measurement of the fluid's interfacial properties is thus essential to better predict the insect's adhesion, and will be a subject of future studies. Further, because of surface inhomogeneity, it may be that not all the hairs are able to completely drain the interfacial water layer, in order for the tarsal adhesive fluid to make direct contact with the substrate. This can further reduce underwater adhesion, in comparison to our theoretical predictions which assumes a perfect contact of all hair terminals.

In the model, we assumed that all the hairs detach simultaneously to give a theoretical maximum achievable adhesion force. In our experiments, however, not all hairs made perfect contact with the substrate despite our best efforts to align the pad parallel to the surface. Furthermore, during detachment, the constrained pad typically peels off from its proximal to distal end rather than detaching simultaneously. Our model also assumes the hairs to be stiff and of similar geometry, unlike the male beetle's pad, which has a distribution of flat- or pointed-tipped soft hairs. Thus, it is not surprising that the model overestimates the adhesion forces. However, when comparing the adhesion in air and underwater, the effect of pad orientation, peeling, hair geometry or elasticity on adhesion should be similar for the two cases, and thus can be reasonably ignored. The model predictions are of the same order of magnitude as experiments, and the qualitative trend is consistent for both hydrophilic and hydrophobic substrates in air and underwater. Further, sensitivity analysis of the model showed that the relative underwater adhesion when compared with that in air was insensitive to the hair or pad geometrical parameters, which validates the applicability of the model for our choice of parameters (see Supplementary Materials and Methods 5). Interfacial tension influenced the relative adhesion for the underwater: no bubble case, whereas contact area and bubble volume influenced the relative adhesion for the underwater: bubble case, as expected.

Our study provides further validation that capillary forces govern the ladybird beetle’s adhesion, and van der Waals contributions, if any, must be negligible. Further, the capillary forces can even enable ladybird beetle attachment underwater depending on the substrate chemistry. When underwater, without a trapped bubble, the pads adhere strongly to a hydrophobic substrate, but poorly to a hydrophilic substrate, even though the pad shows similarly strong adhesion to the two substrates in air. This effect can be explained by capillary forces and the wetting properties of the fluid. Our preliminary chemical composition analysis of a beetle's tarsal secretions before and after immersing its leg underwater (unpublished data) suggests that the tarsal adhesive fluid does not get washed away when underwater. Therefore, the fluid should be able to form capillary bridges and help mediate adhesion even when underwater.

The presence of interfacial water was expected to cause adhesion loss during underwater contact. However, we found that underwater adhesion is possible even for a hydrophilic surface without a trapped bubble (Fig. 2). This suggests the possible role of interfacial water drainage dynamics on adhesion. The experimental adhesion values lie close to the theoretical predictions, which suggests that the interfacial water is drained out within the time scale of contact (∼4 s). The tarsal fluid would then form capillary bridges in direct contact with the surface and enable adhesion. The details of this drainage mechanism during capillary-mediated underwater adhesion would be interesting to look at in a future study.

**Fig. 9.**
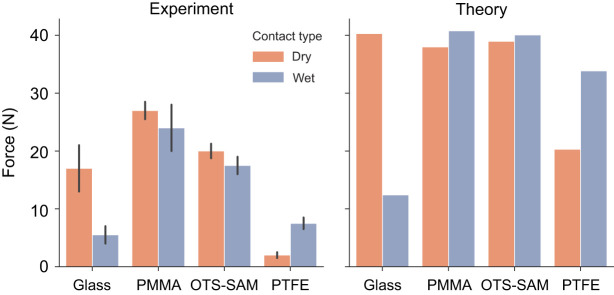
**Whole-animal adhesion force of geckos on various substrates.** Left: experimental shear adhesion values are reproduced from Stark et al. (2013). Right: normal adhesion forces for each gecko toe, theoretically estimated from the capillary bridge model, with hair diameter 400 nm, toe diameter 4 mm, phospholipid fluid volume 4.19×10^−3^ fl and 10% hair coverage. The underwater: no bubble contact mode was assumed for the ‘wet’ case. Net adhesion force was calculated by assuming 5 toes on each leg and 4 legs in total on a gecko. Interfacial tension of the phospholipid layer (PL) in air and water was assumed to be 30 and 42 mN m^−1^, respectively. PL contact angles with glass, polymethyl methacrylate (PMMA), octadecyltrichlorosilane-self assembled monolayer (OTS-SAM) and polytetrafluoroethylene (PTFE) were assumed to be 6, 10, 20 and 80 deg, respectively. The corresponding water contact angles were 50, 85, 94 and 97 deg, respectively, as reported in Stark et al. (2013).

The findings could be extended to some degree to other animals relying on oily secretions for adhesion. For example, ants are known to possess smooth adhesive pads which secrete a fluid containing oily substances (Federle et al., 2002). It has been reported that some ants show similar adhesion on hydrophobic substrates under wet and dry conditions (Stark and Yanoviak, 2018), similar to what we found in a ladybird beetle. This observation can again be explained by a capillary model as before, where the wetting and interfacial tension of the ants' secretion could mediate their underwater adhesion to hydrophobic substrates. Previous experiments on geckos revealed that they can attach well to fluoropolymer substrates (such as polytetrafluoroethylene, PTFE) when underwater, whereas they show little adhesion to the same substrate in air (Stark et al., 2013, 2015). Geckos are thought to rely on van der Waals forces via dry contact with the substrate (Autumn et al., 2002), although observations of phospholipid footprints left behind by walking geckos (Hsu et al., 2012) could change that picture. A recent study has in fact presented evidence for the importance of polar interactions in gecko adhesion mediated by this phospholipid layer (Singla et al., 2021). This calls for a reinterpretation of previously reported gecko adhesion data by considering the influence of the phospholipid layer. In principle, a capillary model could be used to describe the adhesion mediated by this layer, by assuming that the phospholipid compound is mobile with liquid-like properties. As geckos adhere poorly to PTFE (surface energy ∼20 mN m^−1^), one can speculate that the phospholipid material has a higher surface energy, and consequently makes a larger contact angle with PTFE in air. Let us assume the phosopholipid substance to be a fluid similar to oil with γ_fa_=30 mN m^−1^ and γ_fw_=42 mN m^−1^, such that its contact angle with PTFE is 80 deg. Eqn 4 then gives us an underwater contact angle of 70 deg for the phospholipid fluid. Thus, on a PTFE surface, the capillary bridge model can predict a higher adhesion underwater than in air as a result of its lower contact angle and higher interfacial energy underwater. Based on similar assumptions, we can predict the net adhesion force for the gecko on different substrates (Fig. 9). The adhesion force predictions are in good qualitative agreement with the whole-animal experimental shear force values reported for the gecko, with the trend of higher adhesion in air than underwater for glass, similar adhesion in air and underwater for polymethyl methacrylate (PMMA)/octadecyltrichlorosilane-self assembled monolayer (OTS-SAM) and lower adhesion in air than underwater for PTFE. We, thus, propose that the underwater experiments performed on geckos (Stark et al., 2015, 2013) indicate a capillary contribution to gecko adhesion. Previous studies on gecko adhesion have attributed capillary effects to water monolayers adsorbed from ambient humid air onto the spatuale hair tips (Huber et al., 2005; Kim and Bhushan, 2008; Mitchell et al., 2020). However, we emphasise that the capillary contribution in gecko adhesion could instead be a result of its setal phospholipid layer rather than water. The previously reported influence of humidity on gecko adhesion (Huber et al., 2005) could possibly be an effect of change in surface tension of the oily phospholipid layer at different humidity, which will in turn influence the capillary adhesion force. Further work is necessary to understand the details of the mechanism by which the phospholipid layer mediates gecko adhesion.

We have so far limited our analysis to only smooth substrates. Of course insects have to cope with all kinds of surfaces including rough ones. Previous studies (England et al., 2016) have shown that substrate roughness is a more dominant parameter than substrate chemistry in controlling ladybird beetle traction force. Here, the length scale of surface roughness relative to the tarsal fluid thickness would be important in the formation of stable capillary bridges. Further, the presence of an air plastron between the roughness asperities can influence the nature of contact when underwater. Future work will explore how roughness can also impact the net capillary force in wet and submerged conditions. In our study, we have only considered normal adhesive forces, but insects such as beetles in general rely on friction or shear forces during locomotion. Friction force usually correlates directly with the normal force, which is probably why previously reported shear adhesion forces of the dock beetle (Hosoda and Gorb, 2012) follow a similar qualitative trend to our normal adhesion force measurements on the ladybird beetle in both air and underwater conditions. However, the details of the interplay between friction and normal adhesion forces in animals remain unclear and this is beyond the scope of this paper.

Our study can contribute to potential applications in the design of bio-inspired materials to achieve underwater adhesion via capillary bridges. Introduced bubbles can possibly be used to control underwater adhesion by changing the relative proportion of the arrays inside and outside the bubble. A suitable choice of an adhesion-mediating fluid can be made tailored to the substrate and environment of application to form capillary bridges with optimal adhesion performance in bio-inspired fibrillar adhesive systems.

### Conclusions

Ladybird beetles rely primarily on their oily fluid secretion at the tarsal hair tips to adhere to surfaces in both air and underwater conditions. The beetles can attach underwater on a hydrophobic substrate even without a trapped air bubble within the hairy pad, although it loses this ability on a hydrophilic substrate. This is explained theoretically by the different contact angle and interfacial tension of the secreted fluid in air and underwater conditions. Further, the bubble itself has a negligible capillary contribution (less than 3%) to the total force. The trapped bubble can promote adhesion only on a hydrophilic substrate by providing an air medium to the adhesive fluid bridges inside it. Thus, oil wettability primarily controls the insect's adhesion in any given condition. Our study highlights how a fluid-mediated strategy can help achieve strong adhesion even underwater. A similar argument also explains previously reported underwater adhesion force measurements in geckos (Stark et al., 2013), which suggests the possibility of capillary contributions to gecko adhesion mediated by an oil-like phospholipid layer. Future studies should characterise the fluid secretion's interfacial properties with a particular substrate to better understand the fundamental nature of an animal's adhesion.

## Supplementary Material

Supplementary information
