# Supplementary material for: Wetting of the tarsal adhesive fluid determines underwater adhesion in ladybird beetles
Source: J Exp Biol. 2021 Oct 19;224(20):jeb242852. doi: 10.1242/jeb.242852 (PMC8545753; doi:10.1242/jeb.242852)
Supplement: Supplementary information [file jexbio-224-242852-s1.pdf]

Supplementary Materials and Methods 1. Simulation method: Single capillary bridge

Capillary force due to a single adhesive fluid or bubble meniscus (termed “capillary bridge”) was calculated by performing simulations in Surface Evolver[1], similar to the method de- scribed by De Souza et al. A simple cubic geometry, mimicking the capillary bridge, of constant volume, V, was defined as the initial condition with an interfacial tension, γ, with the surrounding medium. Interfacial tension of the capillary bridge with the substrate is given by $\gamma \cos \theta$, where θ is the corresponding contact angle inside the bridge. For the case of a bubble meniscus, θ is defined w.r.t. the surrounding water, since θ can also directly characterise the substrate wettability. The capillary bridge spans a gap distance d between the top face and the substrate. The boundary conditions were set corresponding to a pinned contact line of diameter D on the top face and constant interfacial tension with the substrate

on the bottom. All lengths were normalised relative to length $s = (3V/4\pi)^{1/3}$. An appropri- ate refinement and iteration routine was chosen by trial-and-error to get a stable converged

Journal of Experimental Biology • Supplementary information

solution corresponding to the minimum energy state of the capillary bridge surface. The normalised total capillary force, $\hat{f} = f/\gamma s$, is the sum of the Laplace pressure and surface tension contributions , where:

$$f = f_{laplace} + f_{surface\,tension} = \Delta P_{laplace} A_{bottom} + 2\pi R_{bottom} \gamma \sin\theta \quad \text{(S1)}$$

Here, $\Delta P_{laplace}$ is the Laplace pressure of the equilibrium capillary bridge, A_{bottom} is the contact area of the capillary bridge with the substrate at bottom and R_{bottom} is the corresponding radius of contact, all obtained from the simulation output for the equilibrium surface.

The gap distance d was varied stepwise and the capillary force was calculated each time to obtain force-distance curves for a particular choice of D and θ.

Supplementary Materials and Methods 2. Substrate characterization

The surface chemistry of untreated glass (hydrophilic) and PFOTS-coated glass (hydrophobic) was characterized using dynamic contact angle measurements (Table S1). De-gassed water showed similar contact angle values as normal water.

Table S1. Dynamic contact angles (Mean ± SD, n = 3) of Milli-Q water and n-hexadecane on the different test substrates.

Substrate	Liquid	θ_A	θ_R
Glass	Water	63±5°	20±2°
	n-Hexadecane	<10°	<10°
PFOTS	Water	122±1°	93±2°
	n-Hexadecane	88±2°	56±5°

Supplementary Materials and Methods 3. Statistical comparison

Two-way ANOVA test showed a significant effect of the *Contact mode* (p=0.001, F=9.596, degrees of freedom=2) and *Substrate* (p<0.001, F=36.231, degrees of freedom=1) categories on

Journal of Experimental Biology • Supplementary information

the single leg adhesion force measurements of the ladybug beetle (*Coccinella septempuctata*). Significant interaction between the above two categories was seen (p=0.001, F=10.551, de- grees of freedom=2). Post-hoc analysis results are shown below (Table S2). The uncorrected p-values and Common Language Effect Size (CLES) were obtained from pair-wise Student t-test between A and B while keeping the third parameter fixed (degrees of freedom=8 for each pair). p-values showing statistically significant difference between A and B are in bold- face. CLES represents the statistical proportion of samples under A with higher adhesion than under B. The condition for statistical significance is based on the Bonferroni-corrected critical p-value of 0.008.

Table S2. Post-hoc t-test results for each combination of contact mode and substrate

Fixed variable	A	B	T	p-value	CLES
In air	PFOTS	Glass	-0.053	0.959	0.48
Underwater: bubble	PFOTS	Glass	3.292	0.011	0.96
Underwater: no bubble	PFOTS	Glass	10.044	0.0	1.0
PFOTS	In air	Underwater: bubble	0.133	0.897	0.48
PFOTS	In air	Underwater: no bubble	-0.224	0.828	0.48
PFOTS	Underwater: bubble	Underwater: no bubble	-0.37	0.721	0.44
Glass	In air	Underwater: bubble	4.688	0.002	1.0
Glass	In air	Underwater: no bubble	11.341	0.0	1.0
Glass	Underwater: bubble	Underwater: no bubble	2.086	0.07	0.84

The effect of substrate, contact mode, tilt angle, beetle identity and repetition number on the adhesion were analysed using a linear mixed-effect model (LMEM) in Python. Here, each experimental data point was considered distinctly without averaging the repeats as before. Substrate, contact mode, tilt angle and repetition number were taken as fixed-effects, while,

beetle identity was considered as the random-effect. Interaction between each of the fixed- effects were fitted using the random intercept model. Adhesion measurement on hydrophilic glass *in air* was taken as the reference. The resultant fixed-effects coefficient estimates, standard error, z-statistic and p-value are reported below (Table S3). The random-effect(beetle identity) showed an intercept standard deviation of 100.563 µN (std. error = 109.771)

Table S3. Linear mixed-effect model statistics

	**Estimate**	**Std. Error**	**z**	**p-value**
**Intercept** [1]	582.072	170.307	3.418	0.001
**PFOTS**	-110.642	206.268	-0.536	0.592
**Underwater: bubble**	-304.667	89.458	-3.406	0.001
**Underwater: no bubble**	-254.924	117.386	-2.172	0.03
**Repetition number**	7.723	6.703	1.152	0.249
**Tilt angle**	-5.649	7.088	-0.797	0.425

Supplementary Materials and Methods 4. Capillary force due to an air bubble

Capillary force of a single air bubble against a PFOTS-coated glass surface are compared for two different volumes (Figure S1). The volumes correspond to the expected range for the case of the trapped air bubble in a ladybug's pad. Here, the bubble was pinned to a micropatterned PDMS substrate on the top. Approach-retract tests were performed at 62.5 µm s^{-1} speed. The maximum adhesion force of any of the bubbles never exceeds 50 µN, significantly lower than the beetle's underwater adhesion to the same substrate ($>$ 400 µN). Thus, the bubble's contribution to adhesion in the "*underwater: bubble*" contact of a ladybug's pad should be negligible ($<$ 10 %). Example measurement video is included in the supplementary data (Movie3).

[1] Adhesion on glass *in air*

Journal of Experimental Biology • Supplementary information

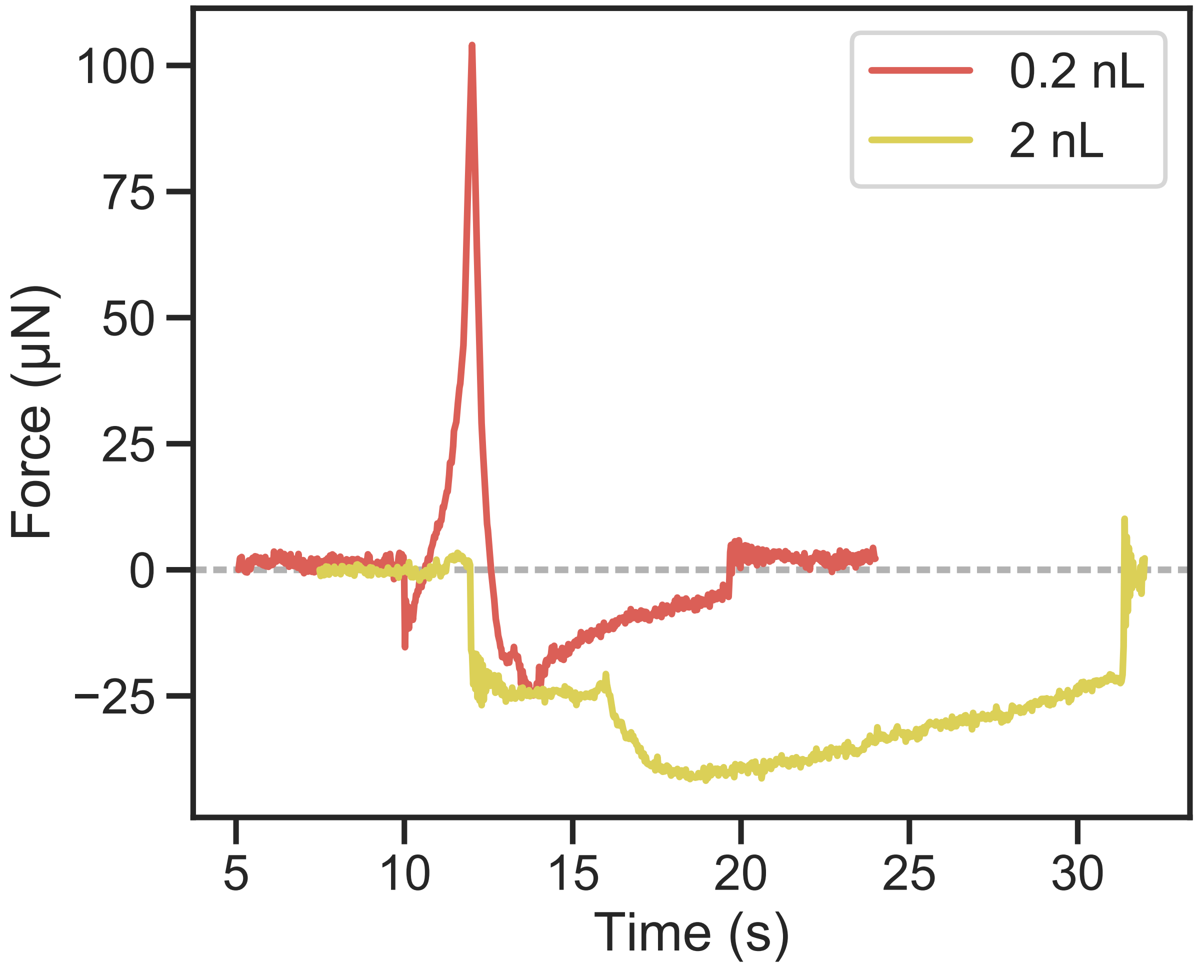

Fig. S1. Capillary force of the pinned bubble against a PFOTS-coated glass surface

Supplementary Materials and Methods 5. Capillary bridge model: Sensitivity analysis

Sensitivity analysis was performed using the one-at-a-time (OAT) method. Dimensionless model parameters were initially set to correspond to the ladybug's case, as given by, contact area fraction ($\alpha = ND_h^2/D_p^2 = 0.1$), pad to hair diameter ratio ($D_p/D_h = 50$), hair aspect ratio ($L/D_h = 10$), water surface tension ratio ($\gamma_{wa}/\gamma_{fa} = 3$), tarsal fluid-water interfacial tension ratio ($\gamma_{fw}/\gamma_{fa} = 2$), tarsal fluid size parameter ($\phi_f = 2$), bubble size parameter ($\phi_b = 1.6$). Substrate contact angles were kept fixed (same as in main text). Each parameter was varied within a particular range, one at a time, and the corresponding adhesion forces *in*

Journal of Experimental Biology • Supplementary information

air (F_a), *underwater: no bubble* (F_w) and *underwater: bubble* (F_b) were calculated. Linear least square regression was performed to quantify the relative change in adhesion for each contact mode with respect to the varied parameter. Here, F_w/F_a and F_b/F_a were taken to be the model output. Slope and R^2 values for each case are reported below (Table S4). Slope with absolute values greater than 0.5 are highlighted in bold.

Table S4. Sensitivity analysis

Parameter	Range	Substrate	F_w/F_a		F_b/F_a	
			slope	R^2	slope	R^2
α	0.05 - 0.3	Hydrophilic	3.03E-18	1.52E-03	2.30E-01	7.72E-01
		Hydrophobic	-9.69E-17	3.03E-03	**-9.40E-01**	7.72E-01
D_p/D_h	30.0 - 60.0	Hydrophilic	-8.83E-20	1.48E-01	1.28E-02	9.73E-01
		Hydrophobic	-5.65E-18	1.48E-01	-1.51E-02	9.82E-01
L/D_h	8.0 - 15.0	Hydrophilic	0.00E+00	0.00E+00	-5.27E-02	9.11E-01
		Hydrophobic	0.00E+00	0.00E+00	5.41E-02	8.66E-01
γ_{wa}/γ_{fa}	2.5 - 3.5	Hydrophilic	-2.01E-01	8.57E-01	-2.43E-01	9.43E-01
		Hydrophobic	4.11E-02	1.00E+00	6.87E-02	1.00E+00
γ_{fw}/γ_{fa}	1.5 - 2.5	Hydrophilic	2.01E-01	8.62E-01	1.90E-01	8.94E-01
		Hydrophobic	**5.56E-01**	1.00E+00	1.57E-01	1.00E+00
ϕ_f	1.7 - 2.2	Hydrophilic	1.29E-02	4.52E-01	6.18E-02	7.94E-02
		Hydrophobic	7.67E-02	9.84E-01	-3.06E-01	9.66E-01
ϕ_b	1.2 - 1.8	Hydrophilic	0.00E+00	0.00E+00	**-1.14E+00**	8.85E-01
		Hydrophobic	0.00E+00	0.00E+00	**1.46E+00**	9.78E-01

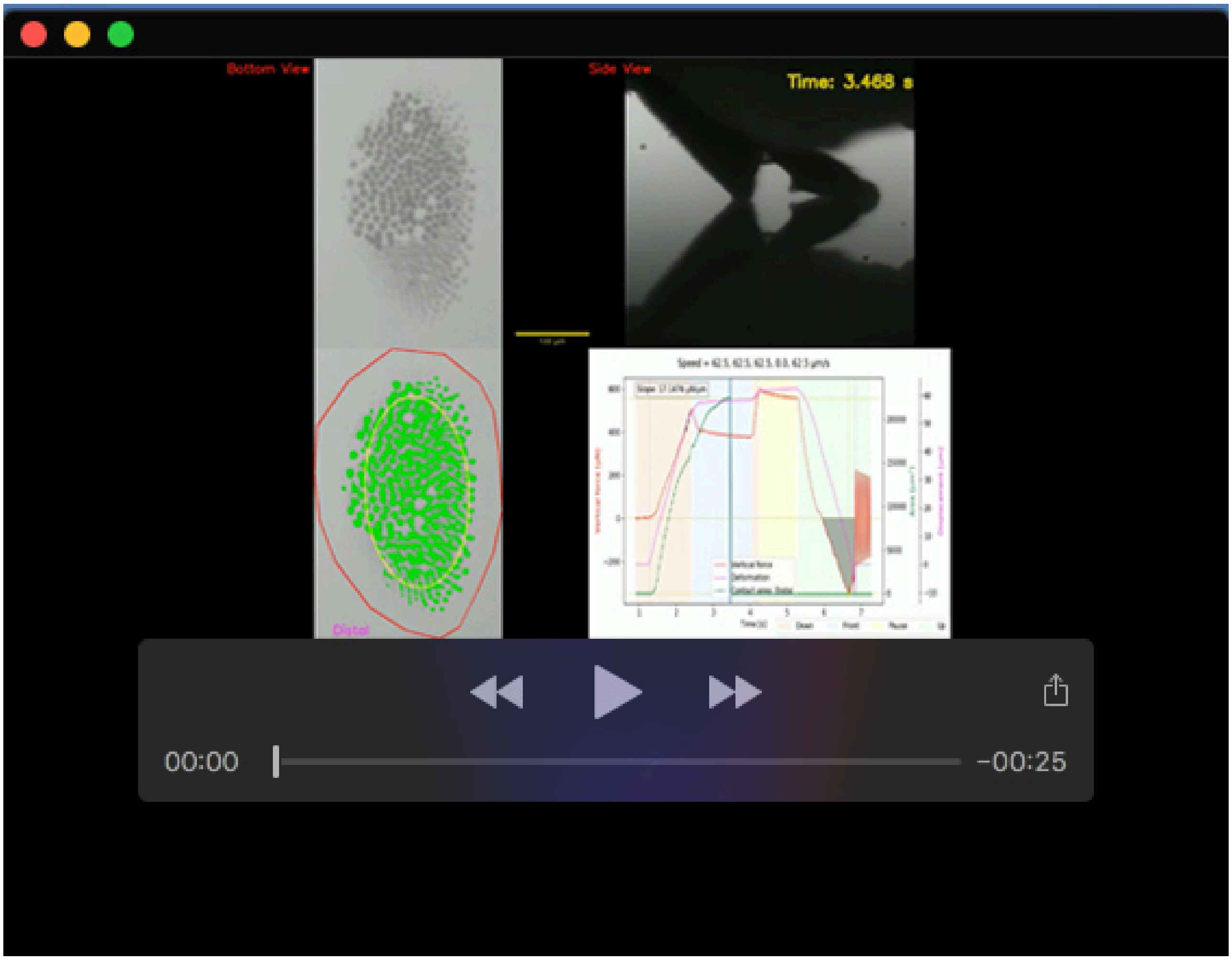

Movie 1. Adhesion test recordings showing the three contact modes: *in air*, *underwater: bubble* and *underwater: no bubble* on a hydrophobic PFOTS-coated glass substrate. The two top panels of the video show the synchronous raw bottom-view and side-view recordings of the pad making contact with the substrate. The lower-left panel shows contact area extraction of the hairs with the surface via image processing and lower-right panel shows the corresponding temporal contact force and area data plot, with the data cursor synchronized with the other panel.

Journal of Experimental Biology • Supplementary information

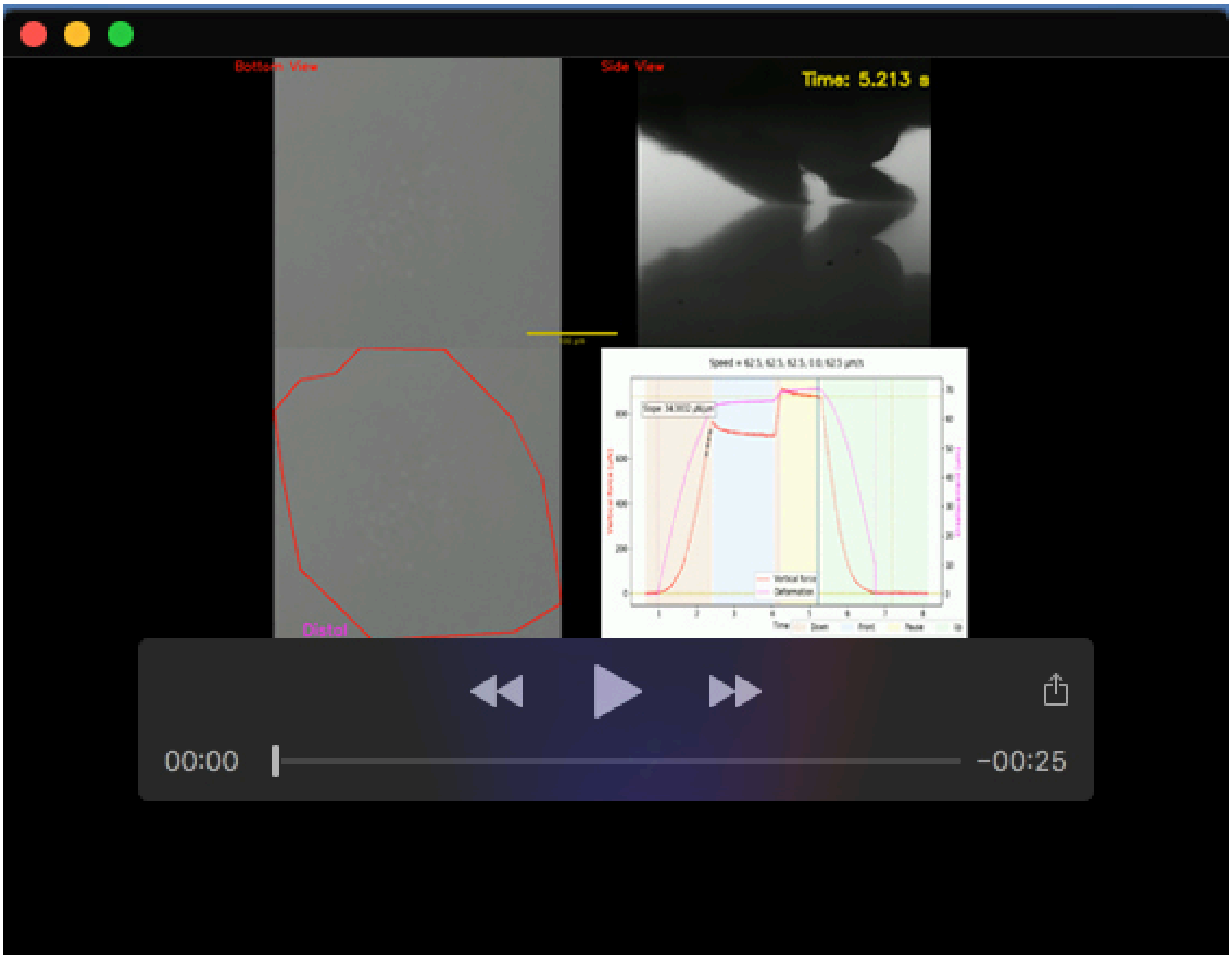

Movie 2. Adhesion test recording corresponding to the case of *bad contact*, which occurred underwateron the PFOTS-coated glass substrate

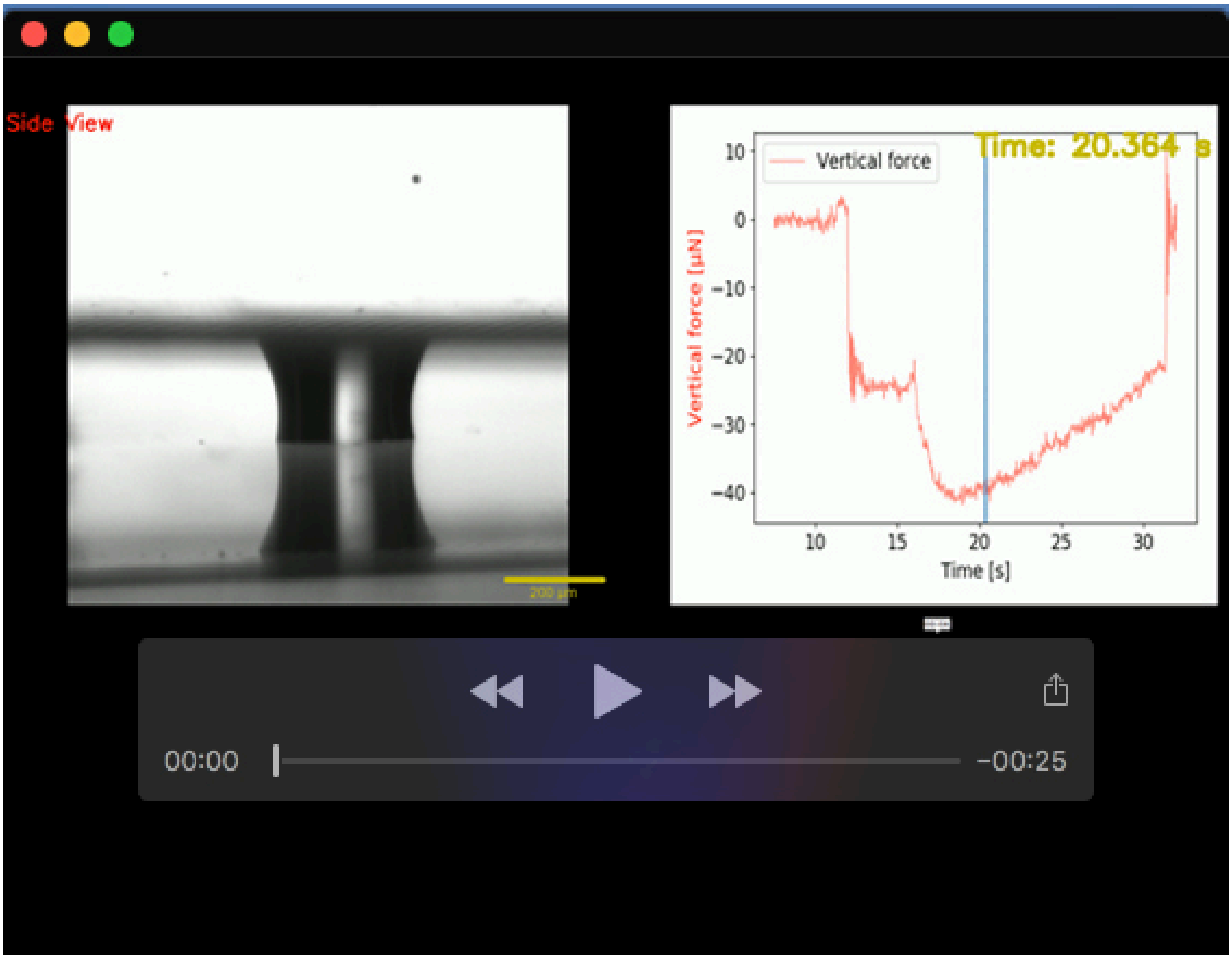

Movie 3. Adhesion test recording of an air bubble (2nL volume) pinned to a microstructured PDMS on the top and making contact with a smooth PFOTS-coated glass substrate on the bottom.

References

(1) Brakke, K. A. The surface evolver. *Experiment.Math.* **1992**, *1*, 141–165.

(2) De Souza, E. J.; Brinkmann, M.; Mohrdieck, C.; Arzt, E. Enhancement of Capillary Forces by Multiple Liquid Bridges. *Langmuir* **2008**, *24*, 8813–8820.
